# Self‐Driving Development of Perfusion Processes for Monoclonal Antibody Production

**DOI:** 10.1002/bit.70093

**Published:** 2025-12-12

**Authors:** Chethana Janardhana Gadiyar, Claudio Müller, Thomas Vuillemin, Jean‐Marc Bielser, Jonathan Souquet, Alessandro Fagnani, Michael Sokolov, Moritz von Stosch, Fabian Feidl, Alessandro Butté, Mariano Nicolas Cruz Bournazou

**Affiliations:** ^1^ Biotech Development Center, Merck Serono SA (an affiliate of Merck KGaA, Darmstadt, Germany) Fenil‐sur‐Corsier Switzerland; ^2^ DataHow AG Zürich Switzerland; ^3^ Chair of Bioprocess Engineering Technische Universität Berlin, Institute of Biotechnology Berlin Germany

## Abstract

The development of autonomous agents in bioprocess development is crucial for advancing biopharma innovation. Time and resources required to develop and transfer a process for clinical material generation can be significantly decreased. While robotics and machine learning have greatly accelerated drug discovery and initial screening, the later stages of development have primarily benefited from experimental automation, lacking advanced computational tools for experimental planning and execution. For example, in the development of new monoclonal antibodies, the search for optimal upstream conditions (such as feeding strategy, pH, temperature, and media composition) is often conducted using sophisticated high‐throughput (HT) mini‐bioreactor systems, while the integration of machine learning tools for experimental design and operation in these systems have not matured accordingly. In this work, we developed an integrated user‐friendly software framework that combines a Bayesian experimental design (BED) algorithm and a cognitive digital twin of the cultivation system. This framework is digitally linked to an advanced 24‐parallel mini‐bioreactor perfusion platform. This results in an autonomous experimental machine capable of: (1) embedding existing process knowledge, (2) learning during experimentation, (3) utilizing information from similar processes, (4) predicting future events, and (5) autonomously operating the parallel bioreactors to achieve challenging objectives. As proof of concept, we present experimental results from a 27 day‐long cultivation including 20‐days operated by the autonomous software agent, which successfully achieved challenging goals such as increasing the viable cell volume (VCV) and maximizing the viability throughout the experiment.

## 1 Introduction

Competition in the biopharmaceutical industry has driven many advances in process development and clinical manufacturing for recombinant proteins. Today, R&D teams are challenged in early development phases to enter clinical trials within very short timelines. During the early phase of molecule screening in drug discovery, different machine learning tools are used at microwell plate (μL to mL) scale to enhance the candidate selection process (Carracedo‐Reboredo et al. 2021; Dara et al. 2022; Khuat et al. 2024; Vamathevan et al. 2019). Following the screening, when the molecule has been identified, bench scale bioreactors (typically 250 mL to 3 L) are used to design the cultivation strategies such as media optimization, feeding strategies and optimal process conditions. For a successful technology transfer to large scale reactors (typically 200L–2000L), a representative scale down high throughput bioreactors is crucial to maximize speed and follow development using Quality by Design (QbD) principles (Anane et al. 2019; Karst et al. 2018). Hence, it is crucial that these scale down reactors have sophisticated monitoring and control systems to emulate the large scale conditions (O'flaherty et al. 2020; Pogodaev et al. 2024). The control system needs to be more advanced for continuous perfusion process with continuous feeds. During the process development of a drug, large number of experiments are carried out in the scale down bioreactor to understand the cell culture process. The process development of the drug will be vastly accelerated by utilization of high throughput scale down bioreactor.

Robotic platforms are used to increase experimental throughput and deliver the best production strategy through QbD approaches (Rouiller et al. 2012; Saleh et al. 2021). There are various examples available in cell line development, upstream process (USP) development and downstream process development. Especially in downstream development, use of robocolumns for screening of resins and process conditions is routinely performed (Baumann et al. 2015). In USP development, microwell plates and automated 2 mL perfusion culture reactors (Mobius Breez, Millipore Sigma) are used to cultivate cells during cell line development and small scale process development activities (Barrett et al. 2010; Bielser et al. 2019; Rouiller et al. 2012; Schwarz et al. 2023). In cell line development, Beacon (Berkley Lights) is an advanced robotic system which can isolate cells in a single pen of 1.7 nL on a chip containing 7000 pens which leads to more efficient clone screening workflows (Le et al. 2020; Rienzo et al. 2021).

**Figure 1:**
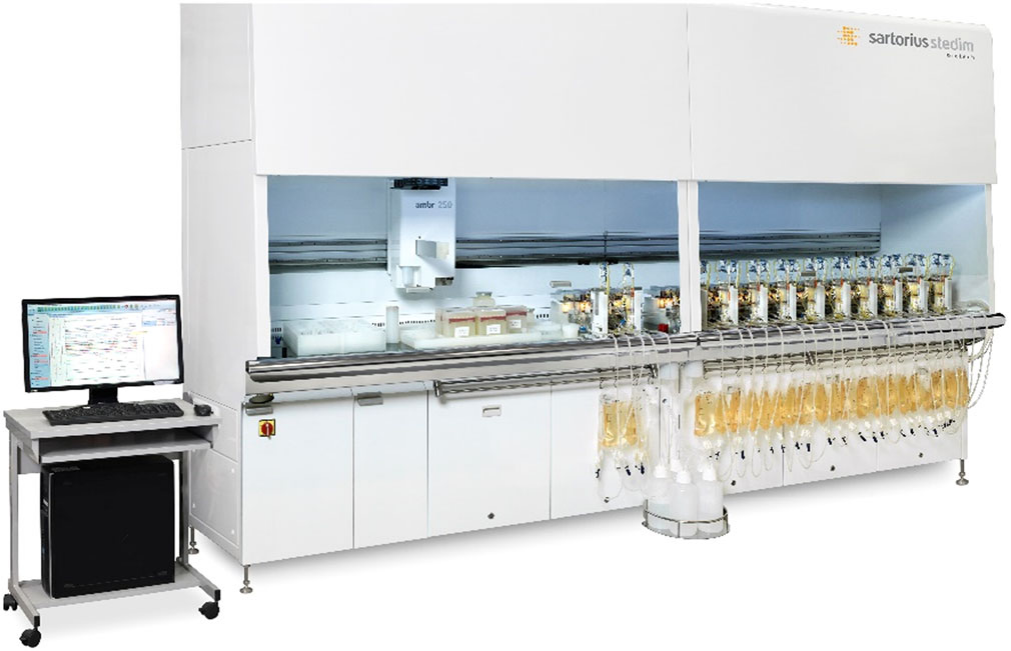
ambr®250 perfusion system.

In the field of cell culture, a highly sophisticated scale down bioreactor is commercially available as ambr®250 robotic platform shown in Figure 1 (Sandner et al. 2019). However, these sophisticated systems do not operate autonomously and require human intervention for design and execution of experiments. For example, perfusion process requires simultaneous control of the media inlet flowrate, the harvest flowrate and the bleed flowrate to maintain a constant volume and cell density. A cell retention device is used to harvest cell free permeate. Despite the high level of automation in these systems, they still heavily rely on manual monitoring and operation. While low level controls (feedings, pH, temperature) are well implemented, computational tools that make important high‐level decisions during operation are currently missing.

Data acquisition also presents challenges, with parameters like pH, dissolved oxygen, and temperature being measured online, while metrics such as viable cell density (VCD), viability, and glucose concentration are assessed at‐line with a frequency of once per day. Furthermore, certain quality attributes may take weeks to quantify (Jang et al. 2009). All these aspects make it difficult for scientists to optimize the experiments while they are ongoing. The robotic platform does not contain the computational tools to support operators to optimize the different process parameters automatically during the run to reach a certain target like high titer and stable quality attributes. The latest review concludes that we lack a comprehensive solution for autonomous operation (self‐driving systems) of parallel cultivation systems with advanced control(Khuat et al. 2024). This is in contrast to the field of chemistry and material science where advanced computational tools are already deployed (Aspuru‐Guzik 2022; Harrer et al. 2023). Self‐driving research is the field where the systems can perform experiments with minimum human intervention by learning from the past experimental results and taking decisions about how to continue the experiments (Abolhasani and Kumacheva 2023). While self‐driving clearly suggests an association with mobile systems the use cases of self‐driving laboratories rarely deal with dynamic systems and the challenges associated with it. Breakthrough results were demonstrated in the field of chemistry, catalysis, material science, and biology (Boiko et al. 2023). For example: over 17 days of continuous operation in which the A‐Lab realized 41 novel compounds from a set of 58 targets including a variety of oxides and phosphates (Szymanski et al. 2023). However, process dynamics become increasingly relevant as we approach industrial scale production (Haringa et al. 2018).

To develop self‐driving systems for efficient bioprocess development and scale‐up, it is essential to incorporate process dynamics and control within this framework (Chopda et al. 2022). We need a digital twin (DT) of the dynamic cultivation process to ensure an optimal design of the experimental campaign and its proper operation by automated systems (Narayanan et al. 2020). Furthermore, due to system uncertainty and variability related to different molecules in biopharma pipeline, the DT requires cognitive properties (Jinzhi et al. 2022). The capability to use existing knowledge and learn from the experiment that is being operated is essential to ensure the fastest development possible and efficient process control of well‐designed experiments (Cai et al. 2020; Kim et al. 2023).

These solutions need to be user friendly to enable its use by experts in experimentation of mammalian processes. Furthermore, the implementation of robust tailor‐made computational pipelines and development of corresponding computational workflows that can automatically handle and store all the data and metadata generated before, during, and after conclusion of an experiment, play an essential role (Bai et al. 2022). Unfortunately, speaking of miniaturized bioreactor cultivations, while the experimental tasks have been automated to great extent and the throughput increased significantly in the last decade, less advances have been achieved in embedding automated computational workflows for data management and decision making.

As a result, while modern experimental robots can perform very complex tasks automatically for long periods of time, experts' intervention is still regularly required to analyze the data and to operate the parallel cultivations. This has to be considered in the context of the large experimental time spans (up to months of cultivation) and the high costs of the experiments (consumables and labor). Finally, the high degree of automation of the experimental setup necessitates an integrated decision‐making agent, to minimize the human involvement in these lengthy and time‐consuming experiments. Algorithms that make the best decisions to operate several cultivation processes in parallel maximizing the knowledge derived from such experiments, are pivotal for accelerating development.

In an effort to tackle these issues and significantly reduce time and costs in the development of monoclonal antibody manufacturing strategies, we present a user‐friendly software solution that enables the autonomous operation of the parallel bioreactor system with perfusion membrane modules. The key features are:

1. A hybrid modeling framework adapted to fed‐batch or perfusion operations and trained with a proprietary data set that can describe the process with sufficient accuracy to enable a robust operation.
2. Online model re‐training to enable real‐time learning leveraging all the information that is being generated during experimentation.
3. A framework to build and retrain hybrid models using the historical process development data of past molecule to transfer their learnings to process development of new molecules.
4. Predictive process monitoring enabling different actions such as notifications, alarms and autonomous actions on process parameters based on optimization functions.

The results demonstrate the capabilities of step‐wise Gaussian process models (SW‐GP) to learn from the data, to transfer learnings between different cell lines, and to support optimal experimental design (OED) with Bayesian methods. They also show that the optimal operation can robustly fulfill challenging tasks throughout longer periods of time and maintain critical conditions as is cell viability within specification.

## 2 Materials and Methods

### 2.1 Cell Expansion

Cells were thawed and diluted at 0.30 × 10^6^ cells/mL in 50 mL SpinTube (TPP, Trasadingen, Switzerland). Cells were diluted every 2 or 3 days to 0.30 × 10^6^ cells/mL or 0.20 × 10^6^ cells/mL respectively and using proprietary expansion medium. Cell culture volumes were increased from SpinTubes to 2 L wavebags (Sartorius, Goettingen, Germany). After 2 weeks of expansion, cells were transferred to inoculate the perfusion cell cultures on the ambr®250 (Sartorius, Goettingen, Germany).

### 2.2 Perfusion Process

All experiments presented in this study were performed on an ambr®250 system with 24 bioreactors vessels of 250 mL volume and a membrane module to enable perfusion cultivations (Figure 1).

The perfusion bioreactors were inoculated with cells at the maximum concentration available in 213 mL of final volume. Cells were cultivated in a proprietary chemical defined medium. During the growth phase, the perfusion rate was increased from 0 to 1.3 d^−1^ until reaching the target viable cell volume (VCV) which was calculated according to the following equation,

(1) $$\mathrm{VCV}\left(\%\right)=\frac{4}{3}\;*\;\pi \;*\;{\left(\frac{d}{2}\right)}^{3}\;*\;\mathrm{VCD}\;*\;100$$

where *d* is the average cell diameter and VCD the VCD (10^6^ cells/mL).

The perfusion volume was maintained constant at 213 mL throughout the duration of the run. Once the VCV target was reached, the culture was considered to be in a state of control and the given setpoints for the input parameters were followed. Bleed was triggered if the target VCV was higher than the setpoint. Sample analysis of the bioreactors was performed daily using a Flex2 (Nova Biomedical, Waltham, MA, USA) to count the cells (VCD), measure cell viability (Via), average cell diameter (Diam), quantify glucose (Glc), glutamine (Gln), glutamate (Glu), lactate (Lac), ammonium (Amm) concentration, pH, pO_2_, pCO_2_ and osmolality. Samples for off‐line analysis were taken to quantify monoclonal antibody (mAb) titer, amino acid concentrations and quality attributes (glycans, oxidation and deamidation) of the mAb produced. A glucose bolus was performed if the glucose concentration was lower than 2.00 g/L after the sampling. The feed volume was calculated to replenish glucose to a concentration of 5.00 g/L.

### 2.3 Training Experiment

A perfusion experiment consisting of 24 runs with a fixed experimental design according to Table 1 was performed with one cell line CHO‐K1 Clone A. The purpose of this run was to gather data with sufficient variability for the initial model training using standard process conditions. This experiment is referred to as the training experiment.

**Table 1:** Steady state experimental plan to build a design space where the digital twin will build models.

Parameter	−1	0	1
Perfusion rate (vessel volume per day (VVD, d^−1^))	0.5	1.25	2
Temperature shift after reaching VCV target (°C)	34	35.25	36.5
Stir speed (rpm)	700	1050	1400
Pyruvate feed flowrate (g L^−1^ d^−1^)	0	1	2

Once the VCV target was reached, all parameters gathered in Table 1 were changed according to the design of experiment (Supporting Information S1: Table S01).

### 2.4 Use Case Experiment

The second perfusion experiment (use case experiment) was performed with two cell lines: Clone A (same clone as training experiment) and Clone B (new clone). The clone A and clone B are derived from the same host cell line. Each clone is expressing a specific monoclonal antibody. Another difference between the two clones is that the transgene coding for the protein of interest is randomly inserted in the genome. In the model, we use a categorical entry named ‘Clone’ to differentiate between the two clones.

Once the state of control was reached, the set points for the selected control variable for each bioreactor (shown in Table 2) were set by the software solution. The software solution optimized the set points of the selected control variables to reach an objective for each bioreactor, as shown in Table 2. All other set‐points were kept the same as in the training experiment.

**Table 2:** Clone tested, control variables and output variables for each bioreactor. Variables to be controlled could change in the tested ranges of Table 1 except for stir speed which could evolve between 700 to 1050 rpm as higher rpm was deleterious for cell viability.

Bioreactor no.	Clone	Selected control variable (range of operation)	Objective
1	Clone A	VVD, Temperature, stir speed, pyruvate flow	Target 30% VCV
2	Clone B	Pyruvate flow	Target 1 mM NH_4_^+^
3	Clone B	VVD, Temperature, stir speed, pyruvate flow	Target 98% viability
4	Clone B	VVD, Temperature, stir speed, pyruvate flow	Target 98% viability
5	Clone B	Pyruvate flow	Target 1 mM NH_4_^+^

## 3 Computational Methodology

In this section, we first discuss the communication with the experimental system using a Structured Query Language (SQL) database. We then shortly discuss the computational framework based on hybrid modeling and Bayesian optimization to finally give a detailed description of the experimental setup.

### 3.1 Gateway Connection and Data Storage

As more laboratories adopt HT experimental technologies and the amount of data generated per time increases, systems for HT data treatment and storage are imminent to assure the traceability and provenance of the experiments as well as to keep up with the data generation speed.

**Figure 2:**
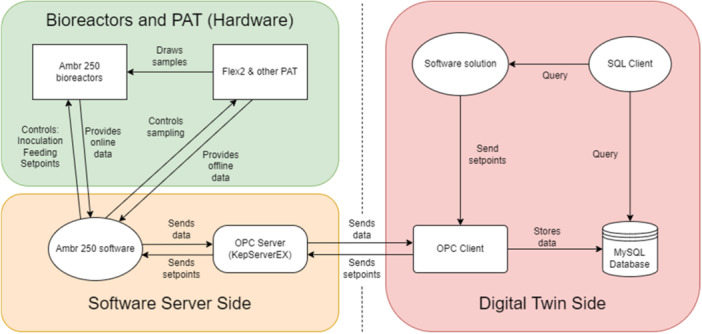
Overview of the interconnectivity of the DT software solution, the OPC and SQL client, as well as the ambr®250 server.

In the current framework, the bioreactor system (ambr®250 bioreactors and the associated PAT shown in green box (Hardware) in Figure 2) interfaces with it's software which is denoted as ambr®250 software (shown in orange box (Software server side). This ambr®250 software interfaces with an OPC UA Server (KEPServerEX) as shown in the orange box (Software Server side) in Figure 2. The data from here transmissions to an OPC client over TCP/IP (developed together with Institutsleiter Elektrotechnik, Hochschule Luzern T&A), to a MySQL Database (shown in pink box (DT side) in Figure 2). This connection collects and stores the data from the ambr 250 bioreactors. The software solution interfaces with the SQL client to query the process data stored in MySQL Database. In addition, the software solution is also able to send optimized set‐points of the control variables to the OPC client, which are loaded to the bioreactors with low level control in real time.

Additionally, all metadata is stored in an Excel macros file (.xlsm) and the actions performed by the bioreactors are recorded and stored in (.csv) files to ensure full traceability during experimentation (Mione et al. 2024). The communication architecture is shown schematically in Figure 2.

### 3.2 Hybrid Gaussian Process Model

The mathematical model used to describe the evolution of the bioreactors during experimentation needs to consider a system with a vast number of cellular biochemical reactions that take place in bioprocess cultivations (González‐Hernández and Perré 2024). In a bioreactor, the chain of reactions that take place in the cells for metabolite secretion and/or antibody production might be unknown. The existing models might not capture all the factors affecting the reactions, for example pH and temperature dependency on shear rates. The presence of impurities in the media can also affect cellular reactions and are very difficult to detect. Due to these reasons, purely mechanistic model might not be able to accurately predict process behavior in a bioreactor without recalibration (Cardillo et al. 2021). Hybrid modelling is an alternative approach, which combines the advantages of mechanistic and data‐driven models to better describe complex systems (Narayanan et al. 2019).

For our current purposes, we use a hybrid model formulation based on basic mass balance equations represented by a system of ordinary differential equations (ODE) combined with Gaussian process (GP) models as the data‐driven counterpart of the hybrid model (De Azevedo et al. 2019; Mahanty 2023). GPs use a measure of similarity (kernel function) between points in the training data to predict the distribution of a value for an unseen datapoint. With the kernel function being usually nonlinear, such an algorithm is capable to reproduce highly nonlinear and complex behavior. The major advantage being the estimation of both, the predicted value and its associated uncertainty (Kocijan et al. 2004).

As reported in literature, Gaussian process state spaces models (GPSSM), that aim to describe the state space with GPs, have shown to also represent mammalian cultivations well (Cruz‐Bournazou et al. 2022; Umlauft et al. 2017). This formulation allows to use GPs to describe nonlinear dynamic processes with limited knowledge of the phenotype and growth dynamics of the cultivation(Hutter et al. 2021). The advantages of GPSSMs have been documented in several applications with small to mid‐size data sets with noise that is close to Gaussian (Kocijan et al. 2005).

Given the slow dynamics of mammalian processes, results show that discretion with a time step of up to 24 h is sufficient to properly mimic the evolution of the process over time. Based on this assumption, we can significantly reduce the computation burden implementing a SW‐GP as described here.

The time discretized ODE system is formulated as:

(2) $$\frac{\mathrm{dc}∙V}{\mathrm{dt}}=V∙\frac{\mathrm{dc}}{\mathrm{dt}}+c∙\frac{\mathrm{dV}}{\mathrm{dt}}≅V∙\frac{c\left(t_{i+1}\right)-c\left(t_{i}\right)}{t_{i+1}-t_{i}}+c∙\frac{\mathrm{dV}}{\mathrm{dt}}$$

(3) $$V∙\frac{c\left(t_{i+1}\right)-c\left(t_{i}\right)}{t_{i+1}-t_{i}}≅R\left(s\right)∙V+u_{f}-u_{b}-u_{p}-c∙\frac{\mathrm{dV}}{\mathrm{dt}}$$

where c is a vector of concentrations of state variables (e.g., VCD, VCV, Via, cell diameter, glutamine, glutamate, glucose, lactate, ammonium, titer); s a vector of process states (i.e., all time dependent variables, including the concentrations c); V is the culture volume; t is a vector with time stamps $t_{i}$; $u_{f}$, $u_{b}$, and $u_{p}$ vectors of mass feed rates (nonzero only for compounds that are fed), mass bleeding rates, and mass perfusion rates, $c\left(t_{i}\right)$ is the concentrations measured at the step i, and $c\left(t_{i+1}\right)$ the concentration measured at the following step, i+1. For the sake of simplicity, in Equation 3 all quantities on the right‐hand side are evaluated at time $t_{i}$.

Note that the change in bioreactor volume over time, dV/dt can be computed explicitly. Using the discrete hybrid model, called SW‐GP from now on, it is possible to calculate the term $R(s\left(t_{i}\right))$ at every time step $t_{i}$, as all other terms are measured, by rearranging Equation 3:

(4) $$R\left(s\right)=\frac{c\left(t_{i+1}\right)-c\left(t_{i}\right)}{t_{i+1}-t_{i}}-\frac{1}{V}∙\left[u_{f}-u_{b}-u_{p}-c∙\frac{\mathrm{dV}}{\mathrm{dt}}\right]$$

We now can explicitly compute the discrete rate of production for every species at the step i, and relate such term to the state of the process measured at the step i, i.e., $s\left(t_{i}\right)$. In the SW‐GP formulation, this is done using a GP model, i.e.: R(s)≈GP(s). The inputs to the GP are the process states s measured at every time $t_{i}$, while the outputs are the corresponding discrete rates of production, R.

The formulation of the discrete hybrid model of Eq. 2 allows to learn the process evolution in time for each variable in a stepwise fashion. Once the initial condition $s\left(t_{0}\right)$ are defined, where $t_{0}=0$, it is possible to compute the rate of production for each state variable in the vector $c\left(t_{i}\right)$ corresponding to the step $\left(t_{i+1}-t_{i}\right)$ and, using the discretized mass balance above, compute e.g. the value $c\left(t_{1}\right)$ at time $t_{1}$. At this point, the state of the process at the new time, $s\left(t_{1}\right)$, is defined and the procedure can be repeated for all steps.

To increase the robustness of the SW‐GP models against batch‐to‐batch variations, process fluctuations, and measurement noise, we implemented an ensemble comprising 20 submodels. The data set is divided into 20 subsets, with 19 subsets utilized for the training of each individual model. This approach significantly reduces the impact of outliers and random variations, thereby improving the robustness of both the forecasts and the optimization processes. This method has proven to be particularly advantageous in biological processes, where variability between replicates is typically low (Liu and Gunawan 2017).

For each prediction, the sub‐models individually predict the process dynamics, and these predictions are used to calculate confidence intervals. Specifically, the 10th to 90th percentiles of the aggregated predictions provide the 80% confidence interval, allowing for uncertainty estimates. The median prediction is given by the 50th percentile.

The relative root mean squared error (rRMSE) is used for both, training and evaluation of the SW‐GP. The model was evaluated considering its capability to predict the next three daily timesteps, since this is the time horizon considered during operation.

The overall error of the model for a state variable x is given by,

(5) $${\mathrm{rRMSE}}_{x}=\frac{1}{\sigma_{x}}\sqrt{\frac{\sum_{t=0}^{t_{f}-1}\sum_{\begin{array}{c} j=1\\ j\leq t_{f}-t \end{array}}^{h}{\left({\hat{x}}_{t+j,t}-x_{t+j}\right)}^{2}}{n_{\mathrm{points}}}}$$

where *t*_*f*_ is the process duration in days, *t* the current process day from which the model predicts, *h* the prediction horizon of interest (here three time steps), ${\hat{x}}_{t+j,t}$the prediction of state variable *x* from day *t* to day *t* + *j*, *x*_*t*+*j*_ the future observed value on day *t* + *j*, *σ*_*x*_ the standard deviation of the respective X variable and *n*_p_ the total number of points to be averaged over, given by,

(6) $$n_{p}=h∙t_{f}-\sum_{j=1}^{h-1}j$$

### 3.3 Online Retraining of Hybrid Models (Example With In‐Silico Data)

Most applications for model‐based operation of robotic experimental facilities work either offline or decouple the learning phase from the experimental plan. Standard methods for adaptive control still rely on mechanistic models and reliable estimates for the initial parameter values (Krausch et al. 2022). These tools might be clone specific and require modelling expertise for use of these tools. However, a cognitive DT which can learn from the ongoing experimental campaign as well as apply the learnings from the previous clones' experimental campaign is necessary for designing process for a new clone (Hutter et al. 2021). Availability of such a tool for high throughput systems will lead to full utilization of the data generated from the parallel bioreactors for process understanding and lead to better process control.

During the process development of a new clone for perfusion process, the experiments that are designed can be better targeted if

1. we are able to transfer existing process knowledge from similar process and
2. we could recalibrate the model with newly acquired data.

The recalibration of the model with newly acquired data is very important because the perfusion run can be long, lasting several weeks.

**Figure 3:**
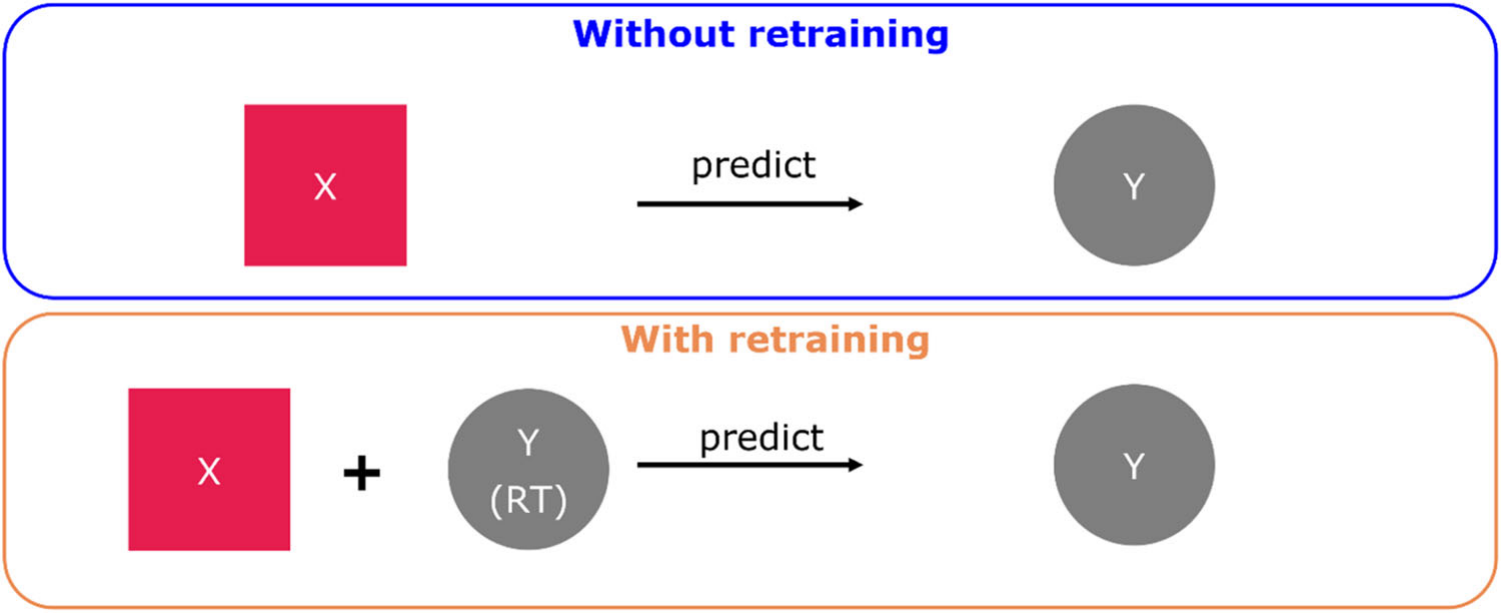
Schematic representation of model comparison approach. In the first case (top row), the model is only trained on clone X and used directly to predict runs of clone Y. In the second approach, the model is initially trained on clone X, but then continuously retrained with current available clone Y data during the process until the process day (RT).

To show the added benefits of online retraining a model during an ongoing experiment, in‐silico datasets for two different clones (clone X and clone Y), corresponding to a data set size of 24 run each, were generated using process experts' knowledge. The data was generated using a mechanistic model that describes fed‐batch cultivation of mammalian processes with lactate consumption (clone X) and without lactate consumption (clone Y). A fed‐batch process over 14 days of cultivation with similar conditions to the use case (see M&M) was considered to generate the insilico data. The state variables are VCD, titer, glucose (Glc), glutamine (Gln), ammonium (Amm), lactate (Lac), dead cell density (DCD) and lysed cells (Lysed). The control variables are the stirring rate set‐point, the DO set‐point, initial conditions of glutamine, glucose and VCD, and the feeding volumes. The hybrid model was initially trained using only data from clone X with 24 runs. The experimental design was simulated with each insilico data point treated as a daily measurement. For the model “without retraining,” only the experimental data set from clone X is used to build the model. For the model “with retraining,” a new model is trained each day using all the data from Clone X as well as process data measured till that day for clone Y as shown in Figure 3. Thus, the model “with retraining” leads to an enhancement of the data set (using new process data from Clone Y) towards the end of the run. This could be beneficial for perfusion runs which typically last for many weeks.

**Figure 4:**
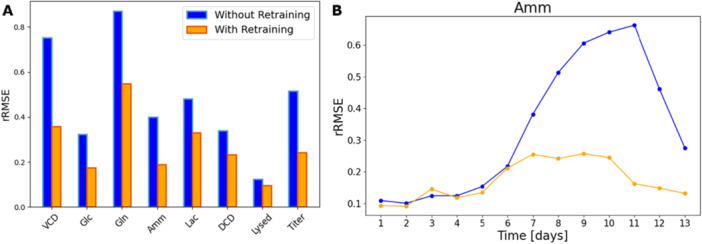
A: Comparison of relative RMSE results when the model is not being retrained (blue) and when it is retrained (orange). Predictions are evaluated over a 3‐day horizon. B: Relative RMSE of Ammonia samples as a function of the process day. Model errors were averaged over all 24 runs in the simulated validation campaign. For the error calculation, only 3‐day ahead predictions were considered until day 11.

Each day, prediction errors were then computed for a 3‐day horizon for both models “without retraining” as well as models “with retraining.” Figure 4A shows the overall error of the model for state variables in the left. The results depicted confirm that, as expected, the model predictions are more accurate in the case that states variables are updated and the model parameters are re‐trained continuously. Detailed analysis of each state variable is described and the overall error of the model calculated each day for all the state variables are shown in Supporting Information S1: Figure S1. We see a trend of lower overall error of the model for the state variables for “model with retraining” compared to that of “model without retraining.”

Figure 4B shows the overall error of the model calculated on each day for “model without retraining” versus “model with retraining” for Ammonium. The overall error is comparable between the two types of model until day 6. However, the model with training shows a clear improvement in the prediction with consistent low overall error beyond day 6. This trend is indeed exemplified for processes which run for long durations, which is typically this use case targeting perfusion processes.

### 3.4 Optimization Framework

Designing experimental campaigns involving multiple parallel runs, extended cultivation durations, and numerous input variables to optimize poses a significant challenge for OED. OED and optimal experimental operation problems have been tackled for many decades by the Process Systems Engineering (PSE) community and has also rapidly gained popularity in the machine learning community (Franceschini and Macchietto 2008; Rainforth et al. 2023). They are also specifically tailored for bioprocess cultivations (Cruz Bournazou et al. 2017; Kim et al. 2022; Martínez et al. 2021).

A very promising approach with the advent of machine learning is to use Bayesian Optimization to treat the problem as a BED task also with several applications for parallel systems (González and Zavala 2023; Luna et al. 2025; Rainforth et al. 2023).

The optimization framework based on Bayesian optimization (BO) searches for the best experimental strategy within the parallel bioreactor system, aiming to maximize the probability that the clone and operating conditions are found. The BO framework searches operating conditions (in particular, temperature, agitation rate, vessel volume per day (VVD) and pyruvate additions) using the SW‐GP model described in 3.2 to compute the acquisition function.

The objective function is constructed by defining a target value for the state variable to optimize and by considering only the model predictions of the next three daily timesteps. The predictions are utilized to evaluate the performance of the process at the given parameter values aiming to find the optimal inputs to bring the process closest to the defined target.

Mathematically, this is described as,

(7) $$\mathrm{OFV}={\left({\hat{y}}_{t+1}-y_{\mathrm{tar}}\right)}^{2}+{\left({\hat{y}}_{t+2}-y_{\mathrm{tar}}\right)}^{2}+{\left({\hat{y}}_{t+3}-y_{\mathrm{tar}}\right)}^{2}(\mathrm{Eq}.6)$$

where *OFV* is the objective function value to be minimized, ${\hat{y}}_{t+j}$ is the predicted value of the process variable *y* at *j* days in the future and $y_{\mathrm{tar}}$ is the desired target value of *y*. The inputs that were chosen to be varied were the set‐points of temperature, agitation rate, VVD (perfusion rate) and pyruvate addition.

### 3.5 User Interface

Finally, to enable a human centric digitalization of the development process in compliance with bioprocessing 5.0, a user interface was developed together with expert users and technical operators (Xu et al. 2021).

**Figure 5:**
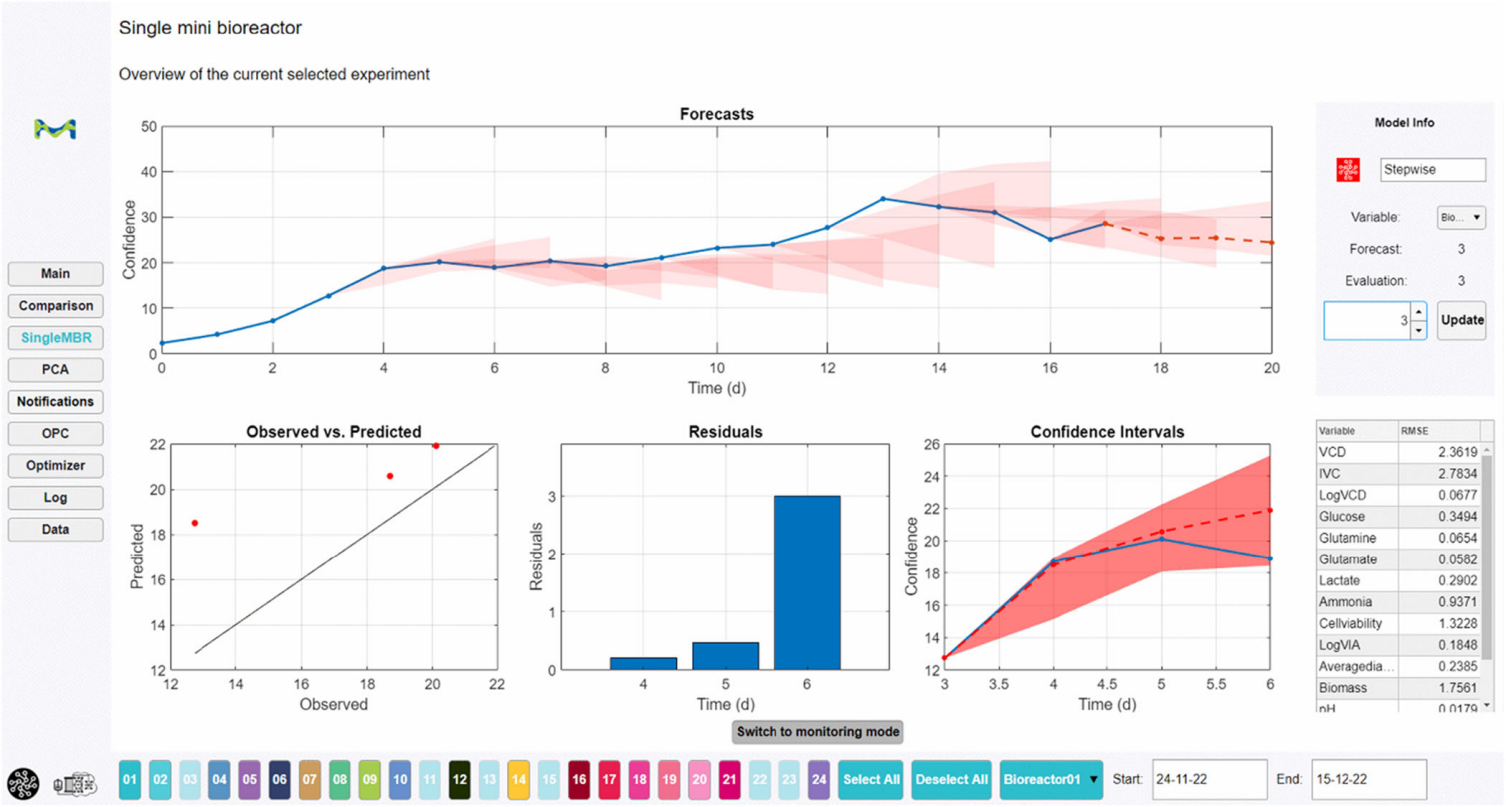
Model evaluation for a single run. Past forecast's prediction intervals are shown in the top plot, as well as current predictions. The visualizations on the bottom show evaluations for a selected process day (here day 3), such as observed versus predicted, absolute residuals and the prediction from that day onward with the prediction interval.

The software solution was equipped with multiple features, which are briefly described below, where some are supplemented with a screenshot of the respective interface:

- Main Overview (Supporting Information S1: Figure S3): Visualization of all measured data for all ongoing bioreactor runs, as well as past experiments. The user may choose any process variable, highlight individual runs or remove them from the visualization.
- Comparison: Multiple univariate data visualizations in one interface, allowing fast comparison across variables and ongoing runs.
- Model evaluation (Figure 5): Overview of all past model predictions for a single run in focus. Includes observed versus predicted for a given process day as well as rRMSE metrics.
- PCA (Supporting Information S1: Figure S4): Multivariate data analysis of all ongoing runs, allowing to identify key correlations between variables, as well as detecting outliers.
- Notifications: The user may set up alarms regarding violation of critical limits of important process variables. The model predicts if constraints will be violated in the future and informs the user on a daily basis if this will be the case.
- OPC: In this section the current connection status is shown and can be queried.
- Optimizer (Supporting Information S1: Figure S5): Here the objective functions for optimization are defined on an individual basis for each bioreactor. The control parameters (such as temperature, perfusion rate, agitation rate and pyruvate addition) are chosen, as well as the lower and upper bounds for search space definition. For the target variables a target value is defined, or they are simply maximized or minimized.
- Log: Record of all actions taken by the software, such as data queries, written set‐points and connectivity status.
- Data: Tabular visualization of all process data of ongoing or past runs.

## 4 Results

### 4.1 Experiment for Initial Model Calibration

**Figure 6:**
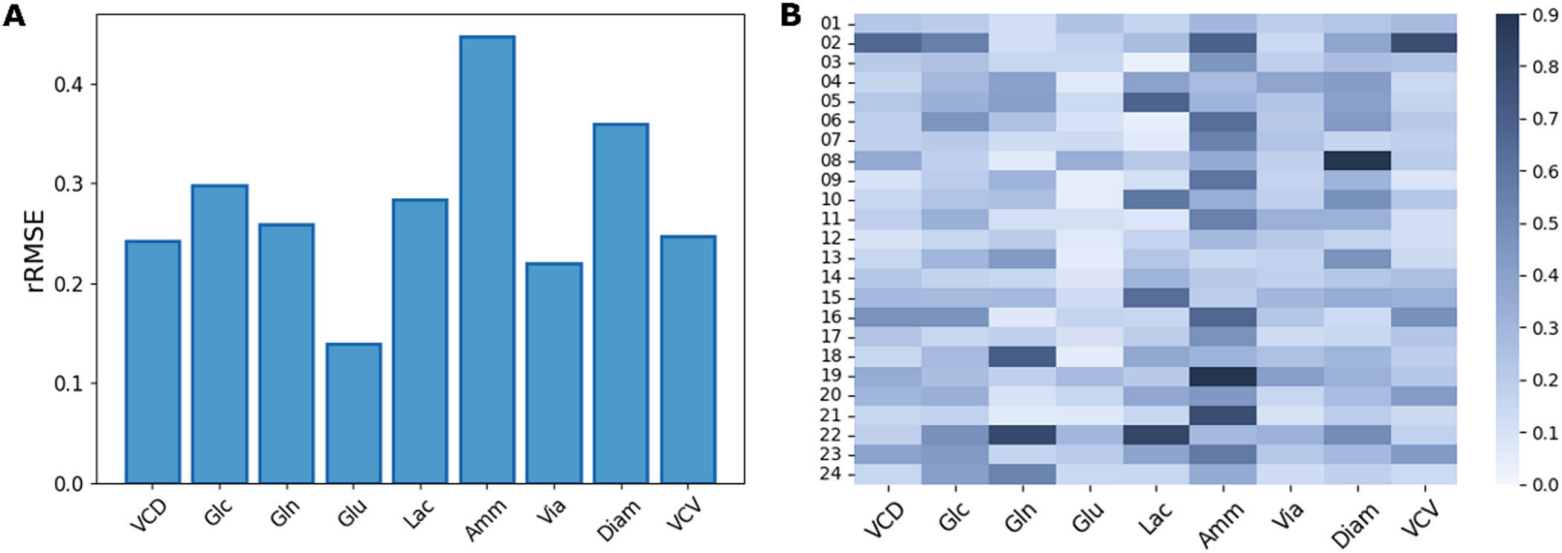
(A) rRMSE results averaged over all runs in testing (leave‐one out) over a 3‐day prediction horizon. The relative RMSE is well below 0.5 for all variables indicating good model performance. (B) rRMSE heat map showing the errors for each run (row) and variable (column).

Specific experiments were tailored to collect data for the perfusion process, as described in Section 3.3 and a hybrid model was trained as explained in Section 2.2. The model output accuracy was assessed by training models in leave‐one‐out validation. The model errors were determined as described in Section 2.5. Figure 6 shows the rRMSE obtained for the trained model against the validation data sets. The left bar plot shows the average rRMSE over all runs in testing over a 3‐day horizon. For all variables the predictions have a rRMSE below 0.5 in the 3‐day prediction horizon considered, making it suitable for control. On the right‐hand side the rRMSE is displayed at the resolution of the runs for each variable. There are no runs (rows), where all variables are predicted with significantly higher rRMSE than the overall results, as can be seen by the intensity of the blue shadings. Therefore, the heat map is indicating that the model can generalize well on the entire design space.

**Figure 7:**
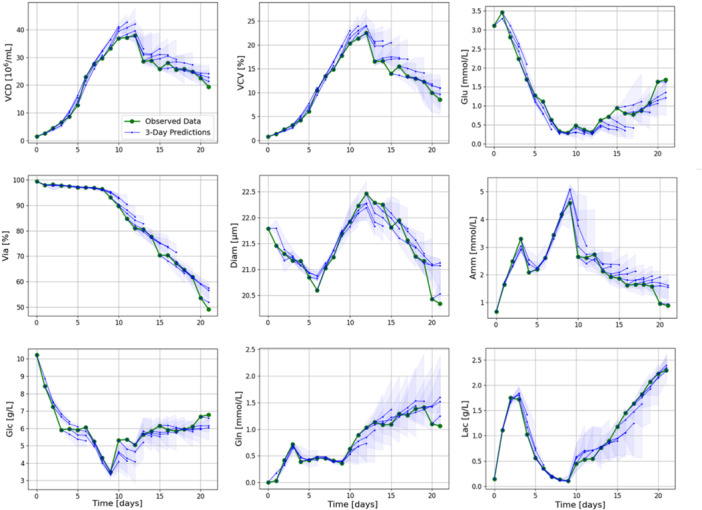
Predictions for a run in the Training experiment in testing. The model was trained on the remaining 23 runs. The green line shows the measured experimental data, the solid blue lines are the 3‐day median predictions of the model, starting on each respective day. The shaded polygons represent the prediction intervals. In the evaluated 3‐day horizon, the model predicts the process trends with sufficient accuracy for software operation.

To better visualize the model prediction capability, a representative run in terms of model performance (Figure 6A) was picked for the purpose of illustration. Figure 7 shows the median model prediction and the prediction interval for the following 3 days on each day of operation. We can clearly see that the model predicts the trends of the process accurately and in general the observed process data is within the prediction interval of previous predictions.

The results demonstrate that the initial calibrated model can learn the process behavior and can make meaningful predictions across different input process conditions.

### 4.2 Demonstration of Software Performance With Use Case Experiments

The online retraining of the hybrid model that was built in Section 4.1 was used to predict the 3‐day dynamic profiles. The optimization framework was used on these dynamic profile predictions to target 3 use case experiments. Three different objectives were selected to target state variables (such as VCV, Viability and Ammonium) to evaluate the online optimization of the bioreactor's control variables by software solution as well as it's retraining capability. The control variables selected for all use cases are perfusion rate, temperature, agitation rate, and pyruvate addition. A single objective (VCV target, viability target or ammonium target) was selected for each bioreactor. The optimization of the control variables and model retraining was performed once per day after the sampling was performed from the bioreactor to measure the state variables. The optimal set points calculated by the software solution for the control variables were set to the bioreactor.

The different use cases demonstrate that a training experiment which covers the expected variability of the control variables will help the hybrid model to learn the impact of the control variables on the state variables. This knowledge from the model can then be used to operate the process once the desired state of control is reached. Deviations from the state of control can be predicted and avoided with a correct response from the system. This strategy is applicable for process development but could also be useful in manufacturing. Discussions on how to validate such models for GMP applications should help the biomanufacturing community to exploit such capabilities to their full potential.

#### 4.2.1 Use Case 1: Increase of the Viable Cell Volume

The objective was set to increase VCV to a target value of 30% for bioreactor no. 1 (Table 2). All the control variables could be varied in the set range as shown in Table 2 at a frequency of once per day.

**Figure 8:**
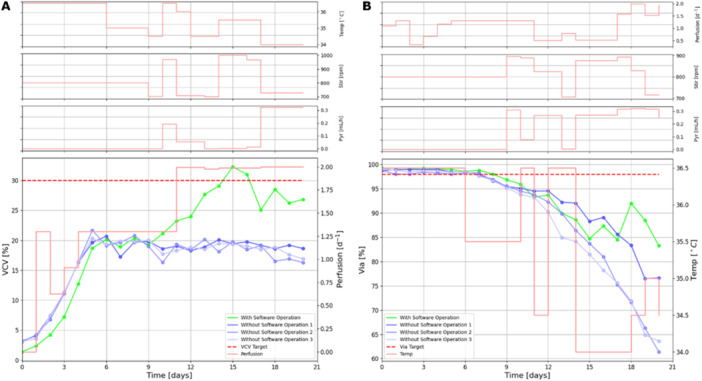
(A) Bioreactor no. 1 results: VCV use case with VCV Target = 30% (red dashed line). The observed VCV values of the bioreactor with software operation are shown in green, the observed values of three bioreactors without software operation are shown in blue. The optimizer suggested perfusion rate is plotted on the secondary axis. All control parameter profiles are shown in light red. (B) Bioreactor no. 3 results: viability (Via) use case with Via Target = 98% (red dashed line). The observed viability values of the bioreactor with software operation are shown in green, three bioreactors without software operation are shown in blue. The optimizer suggested temperature rate is plotted on the secondary axis. All control parameter profiles are shown in light red.

Figure 8A shows the VCV evolution in the bioreactor with software operation (green) and the VCV evolution in 3 bioreactors of the same clone without software operation (blue). Perfusion rate (Perfusion) is the control variable that has the biggest impact on the target and is shown in light red in the large plot. The smaller subfigures at the top of the figure show the set points suggested by the software solution for the other control variables, namely temperature shift (Temp), agitation rate (Stir) and pyruvate addition flowrate (Pyr) respectively.

The software solution was making suggestions from day 9 onwards. For this use case the VCV target value of 30% is significantly higher than the value of VCV in standard experiments (average VCV after state of control < 20%). The software was able to suggest conditions that significantly increased VCV reaching the target in contrast to the benchmark bioreactors.

The software solution identified perfusion rate as the most significant factor for controlling VCV. Higher perfusion rate will lead to faster replenishment of the perfusion media, thereby allowing faster cell growth. This is a well‐known phenomenon among process scientists, which was inherently learned by the hybrid model using the training experiment data as well as the constant retraining of the model.

#### 4.2.2 Use Case 2: Cell Viability

The objective was to maintain the cell viability of clone B above 98% by varying all the control parameters of bioreactor no. 3 as shown in Table 2. Since there exists no previous data on this clone, in the Training experiment, the capability to reach this objective demonstrates the transfer learning capabilities of the approach. Figure 8B shows the cell viability evolution in the bioreactor operated by the software (green) and cell viability evolution in 3 bioreactors of the same clone without software operation (blue). In this use case, temperature is identified as the control variable which had the highest impact on the target and the set points suggested by the software solution are shown in red in the large plot. The daily optimized set points for the other control variables are plotted at the top of the Figure. The results of an additional viability use case are shown in Supporting Information S1: Figure S7.

Similar to the previous use case, the online control of the software solution was started after day 9. The variations in the optimized set points, especially temperature and VVD were quite large until day 14 in comparison to the VCV use case. However, the response of the bioreactor to these changes was used to train the model. The retraining of the model with the new observed data led to an improvement in prediction of cell viability which in turn aids the optimization of control variables. This can be inferred from the increase in cell viability observed after day 15. The software solution suggested lower temperature set points as well as higher perfusion rate towards the end of the process. These conditions led to higher cell viability in the bioreactor in comparison to the bioreactors without software control. Apparently, the ability of the model to understand the impact of the process parameters on viability for the new clone at day 9 was not sufficient. Only after retraining with the data of the first half of the validation run, the optimizer was able to learn these relationships and re‐optimize the set points accordingly.

#### 4.2.3 Ammonium Use Case

It has been suggested in literature that sodium pyruvate addition in a perfusion cell culture can be used to stabilize ammonium concentration (Caso et al. 2022; Romann et al. 2024). Pyruvate is the metabolic entry point for the Krebs cycle. If the glycolysis pathway is saturated and pyruvate becomes limiting, cells will use other sources (amino acids) to feed this cycle, and this can result in ammonium accumulation. Hence, the aim of this use case is to control ammonium concentration to a value of 1.0 mM by using pyruvate addition as the only control parameter. This is illustrated in Table 2 (Bioreactor no. 2) and results shown in Supporting Information S6.

Ammonium concentration increases to 4 mM until day 9 when there is no addition of pyruvate feed. The software solution suggested the addition of pyruvate feed to reach the ammonium target, which led to a reduction in the ammonium value until day 13.

To test the response of the software solution to an increase in ammonium concentration, manual addition of ammonium was done on day 13. Once the concentration of ammonium increased above 7 mM, the software responded by increasing the pyruvate addition set point, as it had learned that the addition of pyruvate decreases ammonium.

The results of two ammonium use cases, with identical targets (Bioreactor no.2 and no.5) and manual injections of ammonium, are shown in Supporting Information S1: Figures S6 and S8.

## 5 Discussion

Novel robotic systems for HT experimentation at every lab scale have drastically changed biotechnology laboratories. The large number of runs that can operate in parallel, the highly complex tasks that robots can perform, the associated process analytical tools and the addition of mobile assistants that overcome spatial limitations, are just some of the many advantages. On the other hand, this significantly increases the number of high‐tech experimental devices that rely on skilled personnel and on a robust digital infrastructure to enable communication, control, and orchestration. There is high reliance on IT systems, large data management tools, experimental design and scheduling optimization, process monitoring and control tools.

As demonstrated in this study, the combination of computational tools with existing robotic experimental setups can drastically increase the throughput and efficiency of laboratories. The online optimization of experimental set points to reach a target objective on a parallel perfusion experiments tackles a major bottleneck in bioprocess development. Moreover, the ability to continuously learn and adapt the experimental design during cultivation allows for real‐time improvements as insights into the process are acquired. This is especially true for development of perfusion processes that are expected to run for long periods of time. A repeated improvement of the experimental design is essential to maximize efficient use of time and resources.

We operated the prototype in a Good Research Practice (GRP) environment, and hence it needed a human intervention for validation of set point changes. This prototype was developed as a proof of concept (POC) which showed promising results and additional work is required to validate it and use it in a Good Manufacturing Practices (GMP) environment.

The use cases presented in this work show that the software fulfills the 5 main requirements for self‐driving laboratories for bioprocess development, namely:

1. **A robust and flexible Hybrid modelling framework for bioprocess:** A robust model that allows a proper description of the time evolution of performance even for new clones is the backbone of an efficient DT of the process. The implemented hybrid model based on SW‐GP was shown to describe the process properly as reported in literature (Mahanty 2023). The modelling framework was successfully implemented during the entire duration of the run spanning over 20 days demonstrating its robustness.
2. **Online model re‐training to enable real‐time learning:** Automated re‐training is essential to empower the algorithm with “learning” capabilities. This feature exploits the fact that in parallel experiments the information generated by neighboring bioreactors is very useful for the running system
3. **A toolset to transfer learnings from past projects:** The software was able to describe perfusion runs with a new clone (Clone B), for which no previous data was available. This is possible because the GP Kernel can extract the existing similarities between the new clone and the known one. By this the existing information can be used to drastically reduce the number of experiments required in development.
4. **Predictive process monitoring and notifications:** Despite the significant autonomy of the robotic system, auxiliary resources and external tasks (liquid containers, at‐line analytics, sample handling for off‐line analysis) still need to be performed by operators. In addition, unexpected malfunction in the bioreactors or liquid handlers need to be tackled by experts. Notifications that inform of an increasing probability that undesired events will take place (low dissolved oxygen, low glucose concentration, deviation on the pH set‐point) allow acting on these events preventively.
5. **Autonomous feed‐back experimental operation minimizing the human in the loop:** One of the key advantages of robotics systems is operation 24/7. Processes that run in tightly defined conditions use process control to ensure robust operation, yet in development, neither a good process understanding (typically in formulated as a mechanistic mathematical model), nor a defined operating regime (as in manufacturing) are available. The software agent in charge of the operation of the parallel cultivation system is confronted with the challenging tasks to learn the new process behavior and simultaneously take actions that drive it to the desired targets (Nair et al. 2022). Furthermore, some targets set in the use case (e.g. cell viability) do not have a known input‐output relationship, and it must be “learned” by the software during experimentation. It is hence very relevant to demonstrate that the system was indeed able to find the process conditions that drive the process to the desired objective in all cases.

The above‐described capabilities make this software solution a viable asset for process development, where high throughput systems are used for optimization of process conditions.

## 6 Conclusion

The introduction of autonomous experimental facilities (self‐driving laboratories) in the pipeline of biopharma development has the potential to accelerate the long and uncertain path for drugs to reach patients. The software solution presented here tackles important challenges by exploiting the potential of advanced parallel mini‐bioreactor experimental systems. We demonstrate the capabilities of the developed software with three use cases, showing the added value of the implementation of machine learning tools in modern experimental systems.

An important challenge that remains to be solved in the future is modelling and feedback operation considering product quality. Currently, the main CQAs can only be measured offline leading to a significant time gap between the sampling and data availability (up to weeks). Process Analytical Technology (PAT) tools that enable online or at‐line quantification of glycoforms, HCPs, aggregates, fragments will further boost bioprocess development and operation.

We expect the results to motivate further development in this area and a larger acceptance in the industry. We expect that this will trigger further discussions on model validation for the use of this prototype in a GMP environment in biopharmaceutical industry. The current gap between robotic capabilities and the autonomy of the devices needs to be urgently addressed in bioprocess development for the biotechnological and biopharmaceutical industries to match the high expectations set to digital biotechnology and bioindustry 5.0.

## Author Contributions

**Chethana Janardhana Gadiyar:** writing – original draft, review & editing. **Claudio Müller:** writing – original draft, data preparation, visualization, modeling, software, conceptualization. **Thomas Vuillemin:** writing – original draft, experimental operation, experimental design, conceptualization. **Jean‐Marc Bielser:** writing – original draft, review and editing, project lead, conceptualization. **Jonathan Souquet:** writing – review and editing, funding acquisition. **Alessandro Fagnani:** writing – original draft, data preparation, modeling, software, conceptualization. **Michael Sokolov:** writing – review and editing, modeling, funding acquisition. **Moritz von Stosch:** Writing – review and editing, modeling. **Fabian Feidl:** writing – review and editing. **Alessandro Butté:** writing – review and editing, modeling, funding acquisition. **Mariano Nicolas Cruz Bournazou:** writing – original draft, supervision, project lead, conceptualization, funding acquisition.

## Data Availability Statement

All data used in this study can be made available upon request.

## Supporting information

SI Self driving development of perfusion process for mAb production.
