# Supplementary material for: Self‐Driving Development of Perfusion Processes for Monoclonal Antibody Production
Source: Biotechnol Bioeng. 2025 Dec 12;123(2):391–405. doi: 10.1002/bit.70093 (PMC12779186; doi:10.1002/bit.70093)
Supplement: Supplementary file 1 — SI Self driving development of perfusion process for mAb production. [file BIT-123-391-s001.docx]

Self-driving development of perfusion processes for monoclonal antibody production

Chethana Janardhana Gadiyar^1^, Claudio Müller^2^, Thomas Vuillemin^1^, Jean-Marc Bielser^1^, Jonathan Souquet^1^, Alessandro Fagnani^2^, Michael Sokolov^2^, Moritz von Stosch^2^, Fabian Feidl^2^, Alessandro Butté^2^, Mariano Nicolas Cruz Bournazou^2,3^

^1^Biotech Development Center, Ares Trading SA (an affiliate of Merck KGaA, Darmstadt, Germany), Fenil-sur-Corsier, Switzerland

^2^DataHow AG, Zürich, Switzerland

^3^Technische Universität Berlin, Institute of Biotechnology, Chair of Bioprocess Engineering, Berlin, Germany

Corresponding author: Mariano Nicolas Cruz Bournazou, mariano.n.cruzbournazou@tu-berlin.de

# Supporting Information


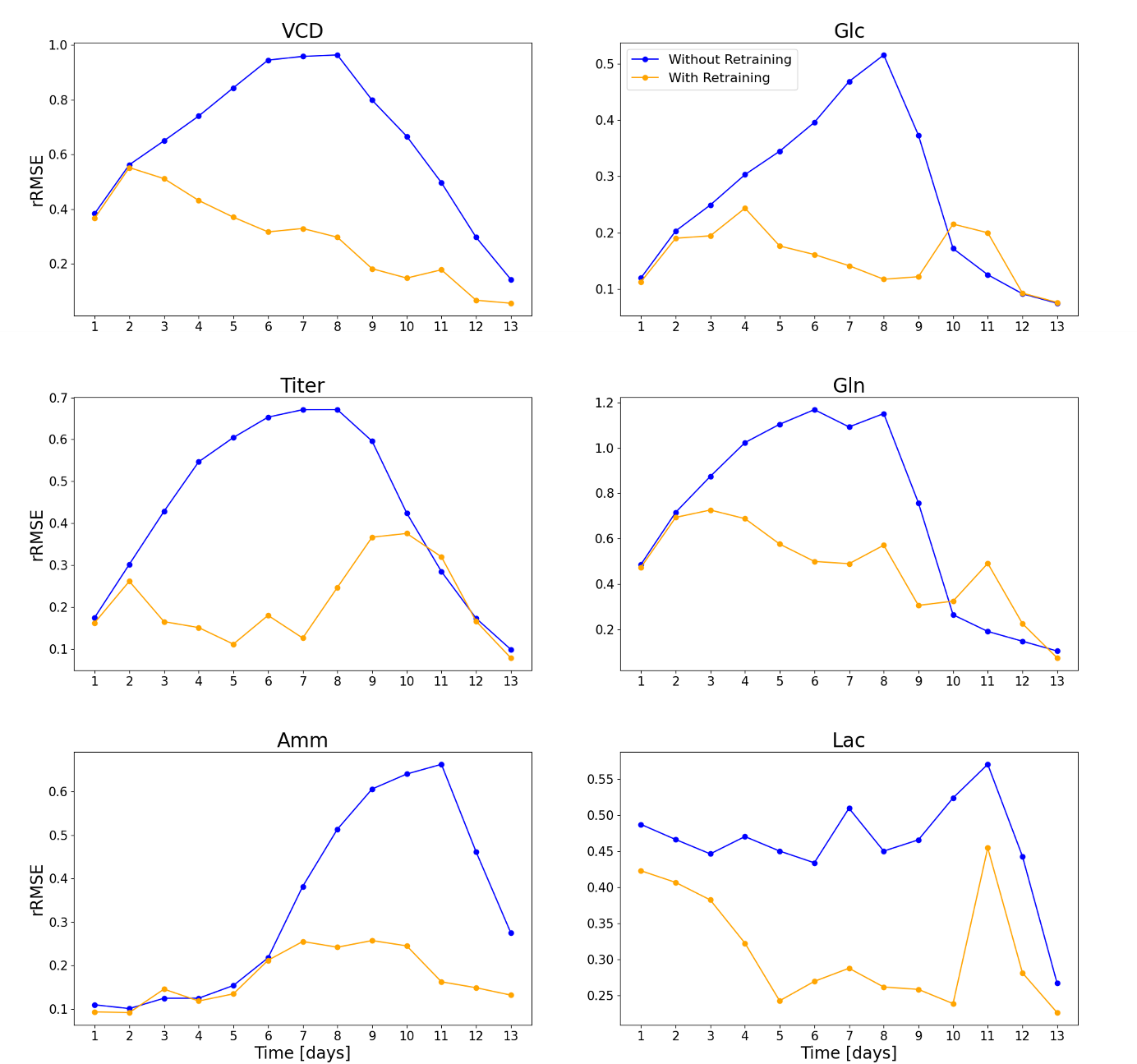


Figure S1 - Relative RMSE as a function of the process day the prediction was made from. Model errors were averaged over all 24 runs in the simulated validation campaign. For the error calculation, only 3-day ahead predictions were considered until day 11, for day 12 the model predicted two more days ahead, and for day 13 the model predicted only one day ahead, as the total process duration was 14 days. The results are shown for VCD, Titer, glucose, glutamine, ammonium, and lactate. The model error for the retrained model are decreasing over time for most variables, while at the same time outperforming the model that is not retrained.

In Figure 4A, when the model is not retrained (blue), the errors for some variables, most importantly VCD, are large (above 0.5). The cell behavior of clone Y is different to clone X in some respects, which the model does not understand. This is due to the lack of experimental data on clone Y in the model. If the model is continuously retrained using new daily measurements, the overall error over the entire process improves drastically for each variable. This shows that the model can learn from the ongoing measured data and adapt its parameters to model the new clone. This is consequential for the model’s ability to be used in optimization of the ongoing process, as without retraining the clone’s sensitivity towards certain parameters might not be accurate enough. Similar to the above conclusion, the error of the “With retraining model” (displayed in orange in Figure S1) is lower than that of “Without retraining model” (displayed in blue in Figure S1) throughout the process, and the divergence usually occurs in 2-6 days. For some variables, notably VCD, the error also consistently decreases with more data being gathered, demonstrating even further the power of retraining the model. For some variables the model error at the end of the process is briefly higher for the retrained model. This coincides with the stopping of the feed on day 10. A possible explanation is that the model with retraining has gathered a lot of data where the feed is active, temporarily becoming worse at predicting the process when there is no feeding. However, as can be seen for glucose, titer, glutamine and lactate, with retraining the model recovers and again predicts better than the model without retraining. The error of the model without retraining also gets lower towards the end of the process. This is expected, as predictions horizons shorten and the process either has stagnated or the cells have died, which is easier to predict for any model. Nevertheless, the model with retraining clearly outperforms the model without retraining throughout the majority of the process duration, especially midway through, where the most critical actions are taken for process control.

To compare the predictions of the model without retraining to the model with retraining, results of a representative run are shown in Figure S2. In Figure S2, the observed data is shown in green; the predictions for the 3 following days calculated on each day of operation (termed as “3-day model prediction”) without retraining are in blue and the 3-day model predictions with retraining are in orange. Consider the case of VCD on the top left. The model without retraining predicts an increase of VCD throughout the process duration, as this is the expected behavior of the clone that it was trained on. On the other hand, the model with training shows better predictions in the 3-day horizon. For VCD it soon learns that this clone grows less than the initial clone, given the current process conditions. Consistently for all variables, while the predictions of the two approaches are similar in the beginning of the process, as not a lot of new data is available yet, towards the end of the process they become more and more different.


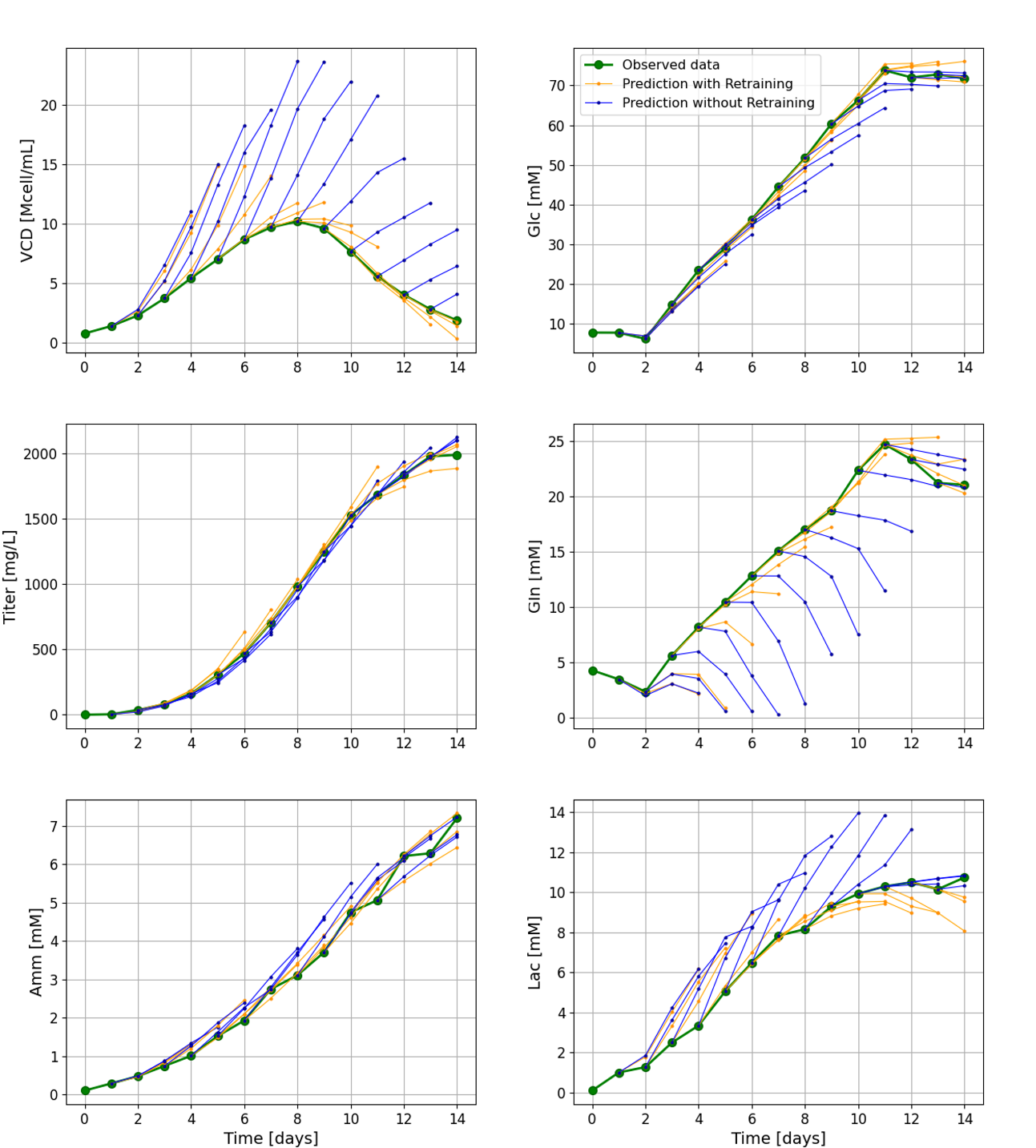


Figure S2 - Observed vs. predicted time evolution profiles for selected process variables, comparing the predictions of the model with retraining (yellow) and the predictions of the model without retraining (blue) to the observed data (green). On each day, the model predicts three days ahead. As can be seen, the model that is continuously retrained learns the behavior of the new clone.


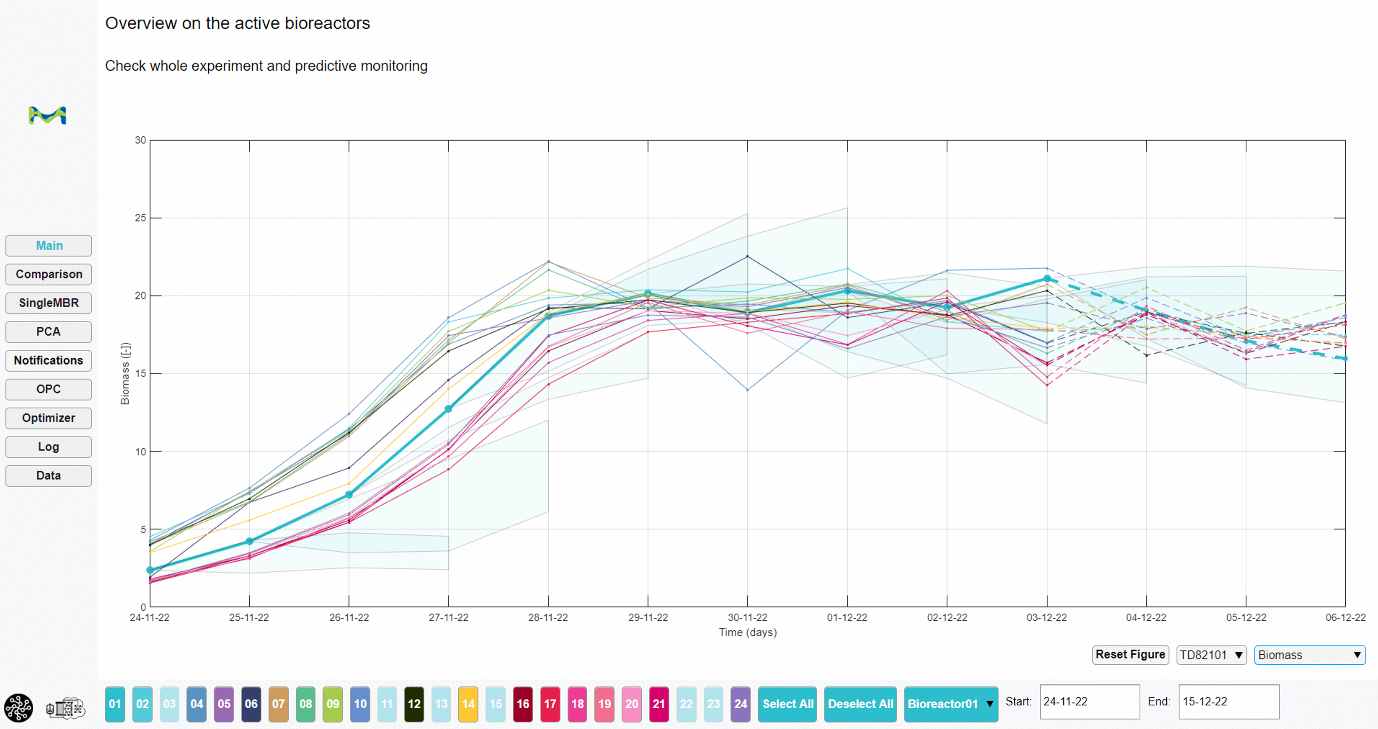


Figure S3 – Main overview of active bioreactors. All process variables can be visualized. The solid line shows the measured process data, whereas the dashed lines that start from the current process day represent the model’s predictions three days into the future. The shaded polygons are the past and present prediction intervals of the highlighted run.


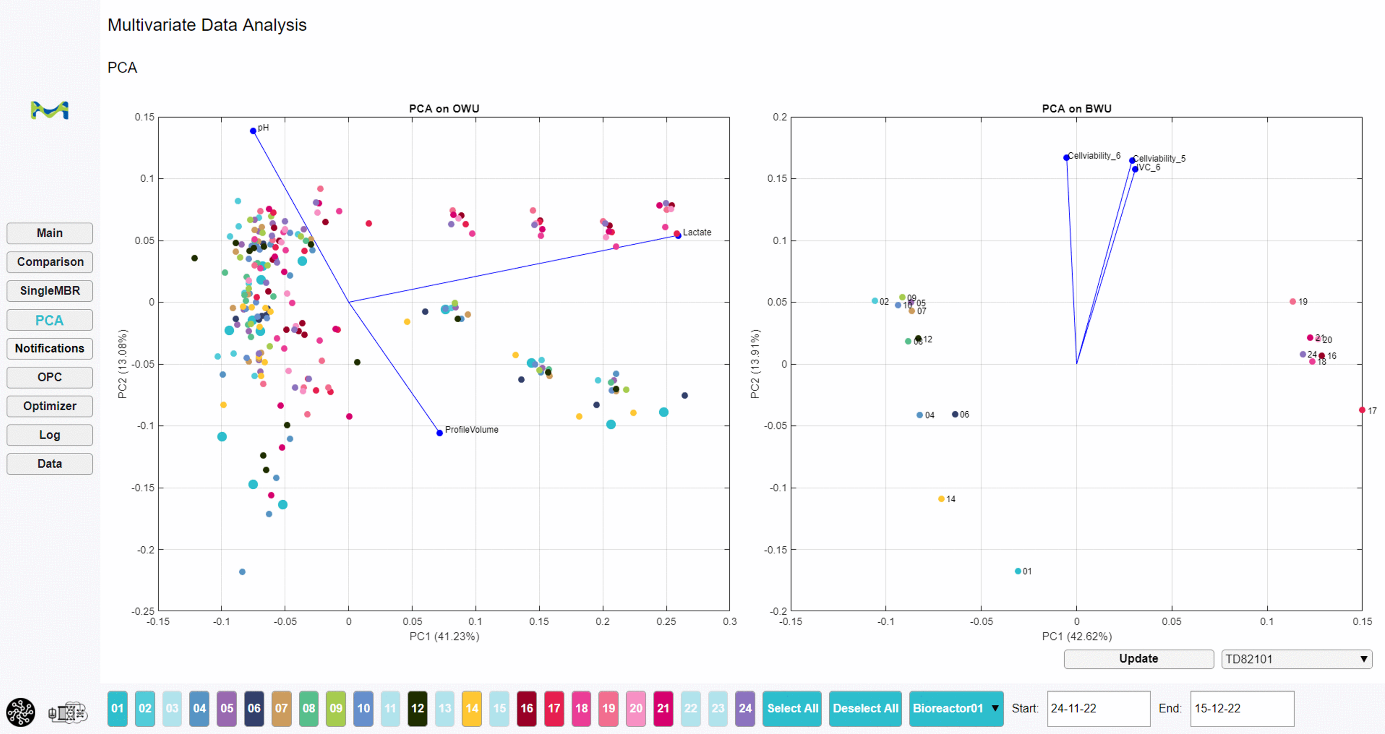


Figure S4 – PCA plots of the variable- or observation wise unfolded (OWU) matrix on the left and batch-wise unfolded (BWU) matrix on the right. Variance explained of the first two principal components are shown, the three loadings with the largest magnitude are highlighted as blue vectors.


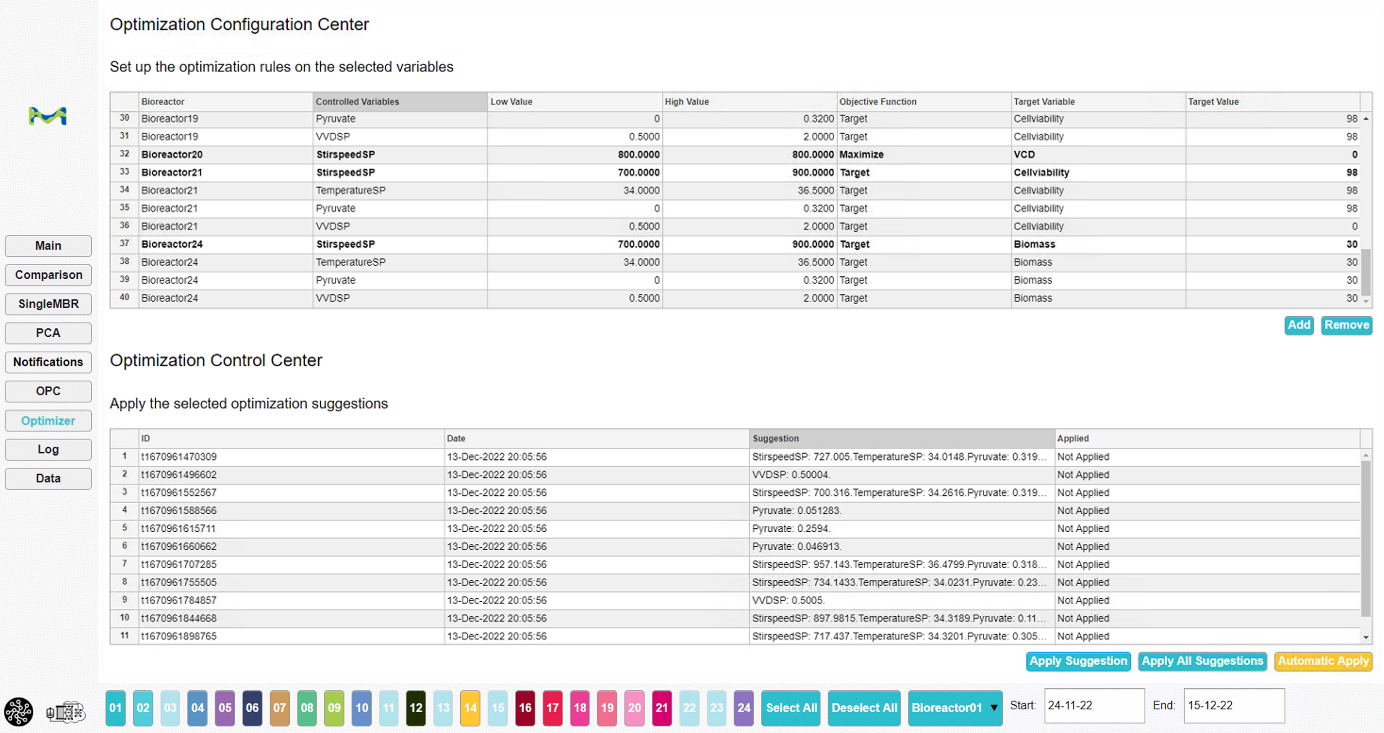


Figure S5 – Optimization configuration center. For each bioreactor, the control variables are configured in terms of lower and upper bounds to define the optimization search space. The target variables can be maximized or minimized, or a specific target value can be chosen. Each day, optimizations are performed upon querying the current data. The software supports manual applying of suggested conditions or an automatic mode.

## Use case experiment results


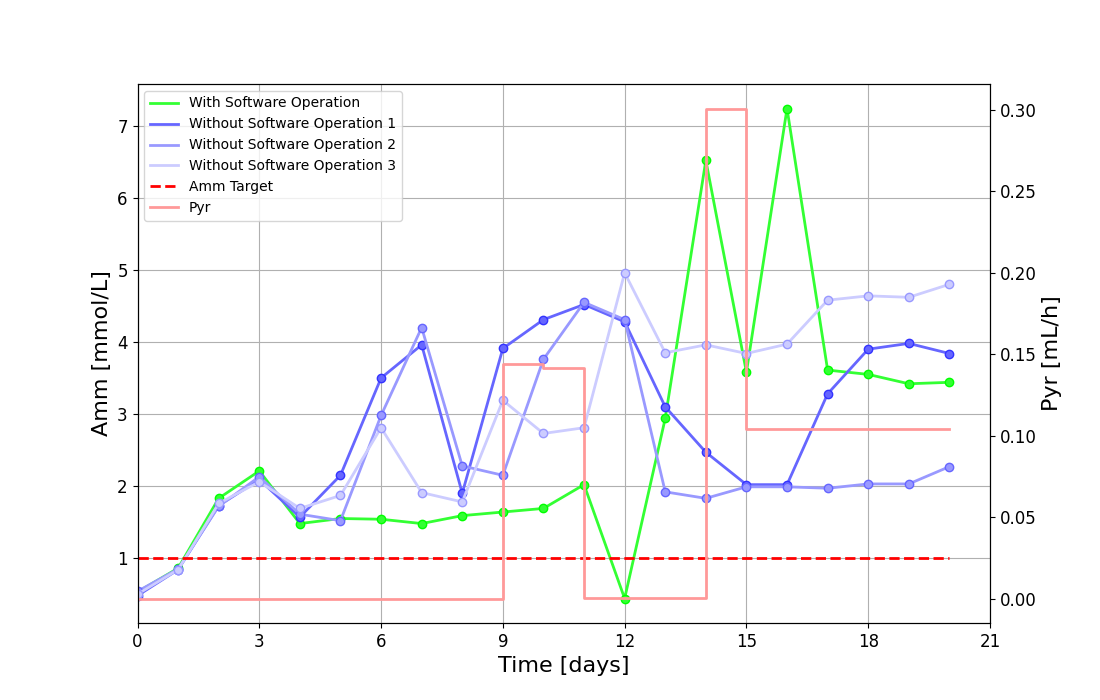


Figure S6 – Bioreactor no. 2 results: ammonium (Amm) use case with Amm target= 1 mM (red dashed line). The observed ammonium values of the bioreactor with software operation are shown in green, three bioreactors without software operation are shown in blue. The optimizer suggested pyruvate additions are plotted on secondary axis and is shown in light red. The model was originally not trained on data of this clone.


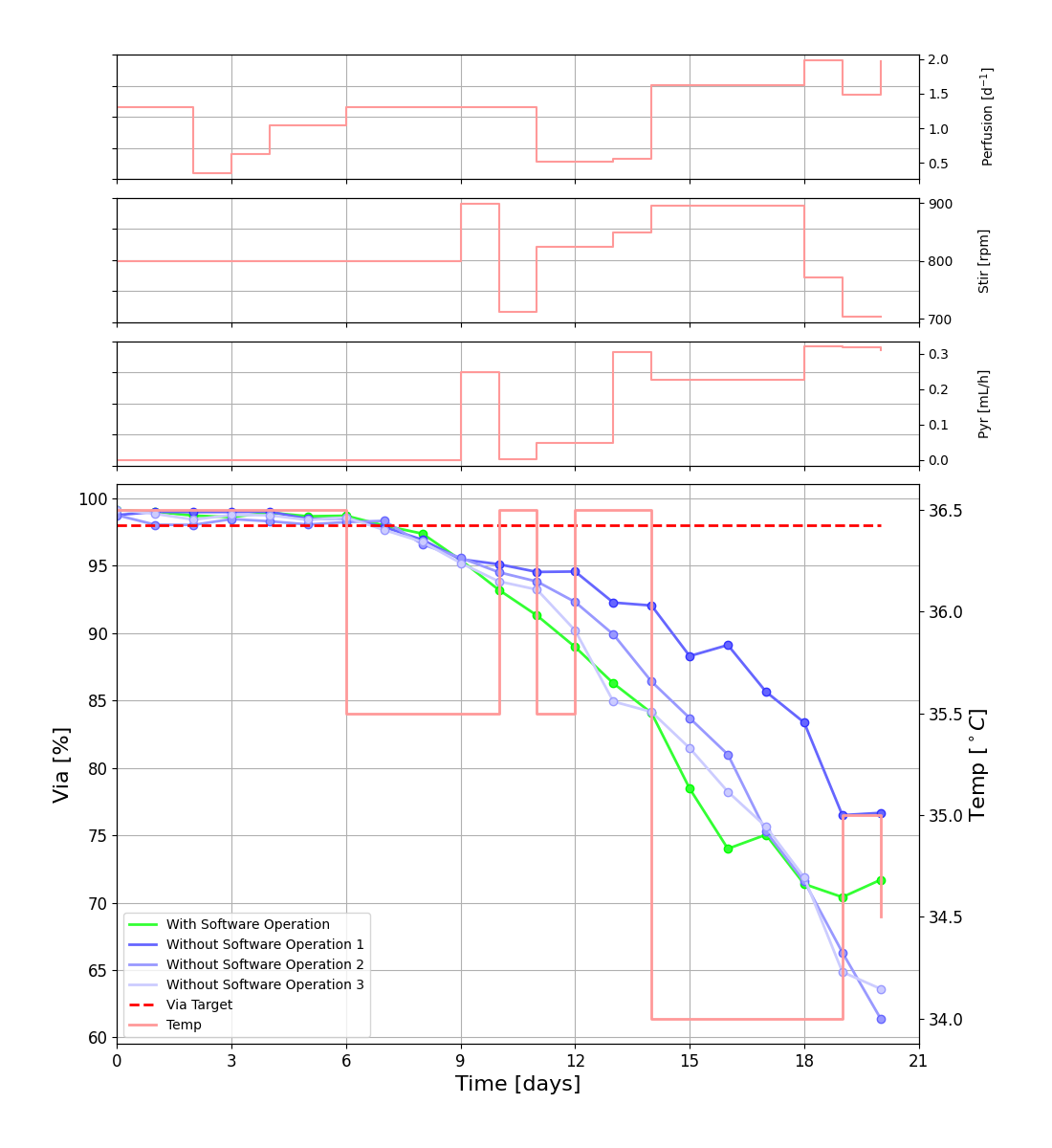


Figure S7 – Bioreactor no. 4 results: viability (Via) use case with Via Target = 98% (red dashed line). The observed viability values of the bioreactor with software operation are shown in green, three bioreactors without software operation are shown in blue. The optimizer suggested temperature rate is plotted on the secondary axis. All control parameter profiles are shown in light red.


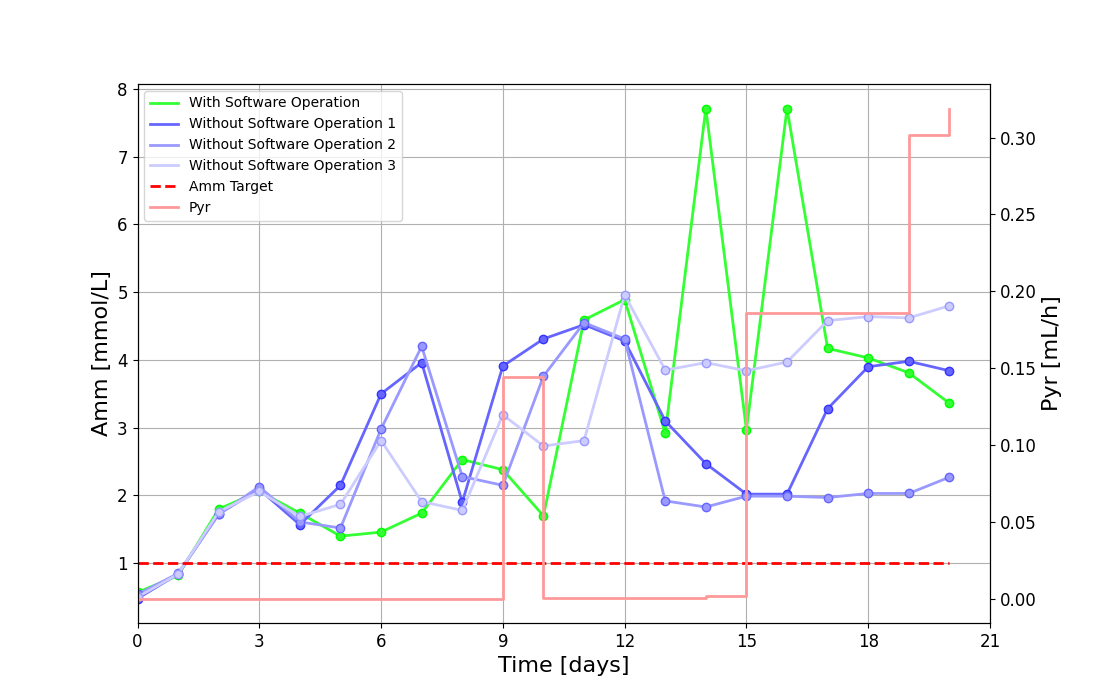


Figure S8 – Bioreactor no. 5 results: ammonium (Amm) use case with Amm target= 1 mM (red dashed line). The observed ammonium values of the bioreactor with software operation are shown in green, three bioreactors without software operation are shown in blue. The optimizer suggested pyruvate additions are plotted on secondary axis and is shown in light red. The model was originally not trained on data of this clone.

| **Vessel Volume exchange per day (VVD)** | **Temperature shift after reaching the biomass setpoint** | **Stir speed** | **Pyruvate flow** |
| --- | --- | --- | --- |
| 1 | 1 | 1 | 1 |
| 1 | 0 | -1 | 0 |
| -1 | -1 | -1 | -1 |
| -1 | -1 | 1 | 1 |
| -1 | 1 | 1 | 1 |
| -1 | -1 | 1 | -1 |
| 1 | -1 | 1 | -1 |
| 1 | 1 | 1 | -1 |
| -1 | -1 | -1 | 0 |
| 0 | -1 | -1 | 1 |
| -1 | 1 | -1 | -1 |
| 0 | -1 | 1 | 0 |
| -1 | -1 | 0 | 1 |
| 1 | -1 | 0 | 1 |
| 1 | 0 | 1 | 1 |
| 1 | 1 | -1 | -1 |
| 0 | 1 | 0 | 1 |
| -1 | 0 | -1 | 1 |
| -1 | 1 | 1 | -1 |
| 1 | 1 | -1 | 1 |
| 0 | 0 | 0 | -1 |
| -1 | 1 | -1 | 1 |
| 1 | -1 | -1 | -1 |
| -1 | 1 | 0 | 0 |

*Table S01: Design of Experiments of the training experiment based on levels explained in Table 1*
